# Minimal endoscopic sphincterotomy with papillary balloon dilation versus endoscopic sphincterotomy for the treatment of common bile duct stones (MARBLE Trial): study protocol for a multicenter randomized controlled trial

**DOI:** 10.1186/s13063-025-09076-2

**Published:** 2025-08-26

**Authors:** Tadahisa Inoue, Akinori Maruta, Junichi Kaneko, Toji Murabayashi, Hirofumi Okuda, Masato Yano, Rena Kitano, Shinya Uemura, Shota Iwata, Shun Futagami, Takuya Koizumi, Tomoya Kitada, Yosuke Ohashi, Yosuke Kobayashi, TTSG Consortium and Collaborators

**Affiliations:** 1 Department of Gastroenterology, Aichi Medical University, Aichi, Japan; 2 First Department of Internal Medicine, Gifu University Hospital, Gifu, Japan; 3 Department of Gastroenterology, Iwata City Hospital, Shizuoka, Japan; 4 Department of Gastroenterology, Ise Red Cross Hospital, Mie, Japan; 5 Department of Gastroenterology, Seirei Hamamatsu General Hospital, Shizuoka, Japan

**Keywords:** Common bile duct stones, Endoscopic sphincterotomy with balloon dilation, Endoscopic sphincterotomy, Endoscopic papillary balloon dilation, Randomized controlled trial, Multicenter study, Adverse events, Stone removal

## Abstract

**Background:**

The first-line treatment for common bile duct stones (CBDS) is endoscopic transpapillary stone removal, typically performed using either endoscopic sphincterotomy (EST) or endoscopic papillary balloon dilation (EPBD). However, EST is associated with risks of bleeding and perforation, while EPBD carries a significant risk of post-procedural pancreatitis. Recently, a combined approach involving minimal EST followed by EPBD (ESBD) has been reported to mitigate these drawbacks, offering potentially safer and more effective outcomes. Nevertheless, no prospective study has adequately evaluated the utility of ESBD, as prior studies were mainly observational or limited by small sample sizes. Therefore, we designed a randomized controlled trial to investigate whether ESBD is superior to EST for the treatment of small CBDS.

**Methods:**

This study is a multicenter, randomized, open-label, parallel-group trial; outcome assessors will not be blinded, but objective predefined criteria will be used to minimize bias. Eligible participants will include patients aged 18 years or older diagnosed with CBDS who require endoscopic stone removal, with eligibility confirmed via imaging modalities. After confirming eligibility, patients will be randomly assigned in a 1:1 ratio to either the ESBD group or the EST group. In the ESBD group, a minimal sphincterotomy will first be performed, followed by balloon dilation for stone extraction. In the EST group, a medium incision extending beyond the hooding fold will be performed prior to stone extraction. The primary endpoint is the incidence of procedure-related adverse events, including pancreatitis, bleeding, and perforation. Secondary endpoints include technical success rate, clinical success rate, procedure time, need for lithotripsy, and stone recurrence rate.

**Discussion:**

This study is the first multicenter, randomized controlled trial to prospectively evaluate the efficacy and safety of ESBD for the treatment of small CBDS. The findings are expected to determine whether ESBD can serve as a new standard therapeutic option compared with conventional EST.

**Trial registration:**

Japan Registry of Clinical Trials: jRCT1040250008. Registered on 21 April 2025. (https://jrct.mhlw.go.jp/en-latest-detail/jRCT1040250008).

## Administrative information

Note: The numbers in curly brackets in this protocol refer to SPIRIT checklist item numbers. The order of the items has been modified to group similar items (see http://www.equator-network.org/reporting-guidelines/spirit-2013-statement-defining-standard-protocol-items-for-clinical-trials/).

**Table:** 

Title {1}	Minimal endoscopic sphincterotomy with papillary balloon dilation versus endoscopic sphincterotomy for the treatment of common bile duct stones (MARBLE Trial): study protocol for a multicenter randomized controlled trial
Trial registration {2a and 2b}.	jRCT1040250008 registered on 21 April 2025
Protocol version {3}	Version 1.1, last updated on 21 February 2025
Funding {4}	This study was supported by internal resources of the Department of Gastroenterology, Aichi Medical University. An application for funding is planned to be submitted to the Japanese Foundation for Research and Promotion of Endoscopy Grant.
Author details {5a}	Tadahisa Inoue^1)^, Akinori Maruta^2)^, Junichi Kaneko^3)^, Toji Murabayashi^4)^, Hirofumi Okuda^4)^, Masato Yano^1)^, Rena Kitano^1)^, Shinya Uemura^2)^, Shota Iwata^2)^, Shun Futagami^1)^, Takuya Koizumi^2)^, Tomoya Kitada^1)^, Yosuke Ohashi^2)^, Yosuke Kobayashi^5)^ 1) Department of Gastroenterology, Aichi Medical University, Aichi, Japan 2) First Department of Internal Medicine, Gifu University Hospital, Gifu, Japan 3) Department of Gastroenterology, Iwata City Hospital, Shizuoka, Japan 4) Department of Gastroenterology, Ise Red Cross Hospital, Mie, Japan 5) Department of Gastroenterology, Seirei Hamamatsu General Hospital, Shizuoka, Japan
Name and contact information for the trial sponsor {5b}	There is no sponsor for this trial.
Role of sponsor {5c}	N/A because there is no sponsor for this trial.

## Introduction

### Background and rationale {6a}

Endoscopic retrograde cholangiopancreatography (ERCP) is the first-line treatment for common bile duct stones (CBDS) [1]. Stone removal is typically performed transpapillarily using a retrieval basket and balloon catheter. Papillary intervention is required to extract stones into the duodenum, as the sphincter of Oddi functions as a mechanical barrier. For small stones (generally ≤ 10 mm), the standard approaches are endoscopic sphincterotomy (EST) or endoscopic papillary balloon dilation (EPBD) [2].

EST involves incising the sphincter to create a wider opening, thereby facilitating stone removal. However, this technique carries risks such as bleeding and perforation. In contrast, EPBD is technically easier to perform and is associated with a lower risk of bleeding and perforation. However, it may impact the pancreatic duct orifice and is associated with a higher risk of post-ERCP pancreatitis. Given the trade-offs of each technique, the choice between EST and EPBD is often based on patient background and endoscopist preference, though EST remains the more commonly used approach.

Recently, a combination technique involving minimal EST followed by EPBD (ESBD)—has been proposed as a way to offset the limitations of EST and EPBD. While conventional EST involves a medium incision that extends beyond the hooding fold, ESBD employs a smaller incision limited to above the fold, potentially reducing the risk of bleeding and perforation, while the subsequent balloon dilation may still ensure adequate orifice opening. Furthermore, ESBD is hypothesized to reduce the risk of pancreatitis compared with EPBD alone by directing the dilation force away from the pancreatic duct and allowing controlled pressure release.

A retrospective study [3] reported in 2020 compared 114 cases of EST and 321 cases of ESBD, demonstrating that the ESBD group had significantly lower rates of requiring multiple sessions (35.1% vs. 12.8%, *P* < 0.001), shorter procedure time for stone removal (31.6 min vs. 25.8 min, *P* = 0.01), and reduced need for mechanical lithotripsy (16.7% vs. 7.8%, *P* = 0.01). The adverse event rate was also significantly lower in the ESBD group (15.8% vs. 4.4%, *P* < 0.001), particularly for bleeding (7.9% vs. 0.9%, *P* < 0.001), without a significant increase in pancreatitis (2.6% vs. 1.9%, *P* = 0.62).

More recently, in 2024, a randomized controlled trial [4] comparing EST and ESBD reported that the primary endpoint, use of mechanical lithotripsy, was significantly lower in the ESBD group (20.0% vs. 4.0%, *P* = 0.028), and procedure time was also significantly shorter (18.5 min vs. 17.0 min, *P* = 0.047). However, the trial was prematurely terminated due to poor enrollment and included only 100 participants (50 per group) instead of the planned 220, which limits the generalizability of its findings.

Given these promising but preliminary data, ESBD is expected to be a valuable technique for managing small CBDS; however, no prospective studies have yet adequately evaluated its safety and efficacy. Therefore, we designed this randomized controlled trial to determine whether ESBD provides superior outcomes compared with conventional EST and to assess its potential as a new standard approach for papillary treatment during endoscopic management of small CBDS. Previous studies have been limited by small sample sizes, single-center designs, and heterogeneous protocols. The present trial addresses these limitations through a larger sample size, multicenter participation, and standardized procedural protocols.

### Objectives {7}

The objective of this study is to compare the efficacy and safety of ESBD and EST in patients undergoing endoscopic transpapillary removal for small CBDSs. The primary aim is to determine whether the incidence of procedure-related adverse events is significantly lower in the ESBD group. Additionally, secondary comparisons will be made regarding outcomes related to stone removal efficacy, such as the proportion of cases requiring mechanical lithotripsy and the overall procedure time. Details of the study outcomes are summarized in Table 1.

**Table 1:** The primary and secondary endpoints in the MARBLE trial

Outcome	Measurement variable/definition	Analysis metric	Time point
**Primary endpoint**			
Procedure-related AE rate	Proportion of patients experiencing procedure-related AEs after protocol intervention	Incidence proportion	Within 30 days from the procedure
**Secondary endpoints**			
Type and severity of procedure-related AEs	Classified using ASGE lexicon; detailed criteria applied for bleeding and pancreatitis (see Notes)	Descriptive	Within 30 days from the procedure
Technical success rate	Completion of ESBD or EST according to protocol	Binary outcome	Day of procedure
Clinical success rate	Complete stone removal in a single session	Binary outcome	Day of procedure
Total procedure time	Time from cannulation to completion of stone removal	Continuous (minutes)	Day of procedure
Time required for stone removal	Time from end of ESBD or EST to completion of stone removal	Continuous (minutes)	Day of procedure
Proportion of cases requiring lithotripsy	Use of mechanical lithotripsy (excluding mechanical tools used without fragmentation), electrohydraulic lithotripsy, or laser lithotripsy	Incidence proportion	Day of procedure
Stone recurrence rate	Reappearance of bile duct stones post-removal	Incidence proportion	5-year follow-up
Time to stone recurrence	Days from successful stone removal to confirmed recurrence	Time-to-event	5-year follow-up
Biliary event rate	Any biliary event (e.g., stone recurrence, cholangitis, cholecystitis, liver abscess, biliary cancer)	Incidence proportion	5-year follow-up
Time to biliary event	Days from successful stone removal to occurrence of any biliary event	Time-to-event	5-year follow-up
**Notes on adverse event definitions** The definitions and severity grading of procedure-related adverse events will follow the ASGE lexicon [7]. Among these, bleeding and pancreatitis are the most anticipated adverse events in this study and represent key safety outcomes; therefore, more detailed criteria are specifically defined for these two events **-Bleeding:** *Intra-procedural bleeding*: requires hemostatic intervention (excluding preventive measures) *Post-procedural bleeding*: confirmed by endoscopy or imaging study such as computed tomography, or inferred from clinical findings such as hematemesis, melena, or a drop in hemoglobin level that cannot be explained by other causes **-Pancreatitis:** The diagnosis is established when at least two of the following three criteria are met: (1) acute abdominal pain and tenderness in the upper abdomen, (2) elevated levels of serum or urinary pancreatic enzymes, and (3) imaging findings indicative of acute pancreatitis [8]			

*AE* adverse event, *ASGE* American Society for Gastrointestinal Endoscopy, *ESBD* minimal endoscopic sphincterotomy with balloon dilation, *EST* endoscopic sphincterotomy

### Trial design {8}

The MARBLE trial is a multicenter, randomized, open-label, parallel-group controlled trial designed to evaluate the superiority of ESBD over EST in terms of adverse event rate among patients receiving endoscopic transpapillary CBDS extraction. Patients diagnosed with small CBDS will be screened based on the inclusion and exclusion criteria. Eligible patients will be randomized at a 1:1 ratio to either the ESBD or EST groups.

The MARBLE trial has been designed and will be implemented by the TTSG, a multicenter consortium for clinical research, consisting of seasoned pancreatobiliary endoscopists from the Tokai region of Japan.

## Methods: participants, interventions, and outcomes

### Study setting {9}

This study is a multicenter collaborative trial that will be conducted at five tertiary referral centers located in the Tokai region of Japan. Specifically, the participating study sites are Aichi Medical University Hospital, Gifu University Hospital, Iwata City Hospital, Ise Red Cross Hospital, and Seirei Hamamatsu General Hospital.

### Eligibility criteria {10}

The inclusion and exclusion criteria are outlined in Table 2. Patients will be eligible for enrollment if they satisfy all of the inclusion criteria and none of the exclusion criteria. Key inclusion criteria are patients aged ≥ 18 years with CBDS confirmed by imaging, requiring endoscopic removal. Key exclusion criteria include stones ≥ 12 mm and previous papillary intervention. Endoscopic procedures for both groups will be performed by study endoscopists at each participating center. To ensure consistency across sites, all operators will participate in pre-study training and harmonization sessions, including review of procedural steps and photographic examples.

**Table 2:** Eligibility criteria for participants with common bile duct stones in the MARBLE trial

Inclusion criteria*
1. Diagnosed with CBDS based on imaging such as CT, MRCP, abdominal ultrasound, or EUS
2. Judged to require endoscopic stone removal
3. Aged ≥ 18 years at the time of consent, regardless of gender
4. Provided written informed consent based on voluntary agreement after receiving sufficient explanation
Exclusion criteria*
1. CBDS with a stone diameter ≥ 12 mm
2. History of gastrointestinal reconstruction other than Billroth I
3. History of biliary reconstruction
4. Coexisting biliary strictures
5. Coexisting biliary tumors, including duodenal papillary tumors
6. Papillary orifice located within a periampullary diverticulum
7. Patients taking antithrombotic agents for whom medication cannot be discontinued or adjusted according to the Guidelines [5, 6] in patients on antithrombotic therapy
8. Bleeding tendency (platelet count < 50,000/mm^3^ or PT-INR > 1.5)
9. Coexisting severe cholangitis
10. Coexisting pancreatitis
11. History of any previous papillary intervention
12. Judged as unsuitable for endoscopic treatment due to safety concerns
13. Pregnant or possibly pregnant
14. Deemed ineligible for the study by the principal investigator or co-investigators

*CBDS* common bile duct stone, *CT* computed tomography, MRCP magnetic resonance cholangiopanreatography, EUS endoscopic ultrasound, PT-INR prothrombin time-international normalized ratio

^*^Eligible patients must meet all inclusion criteria and none of the exclusion criteria to be enrolled

### Who will take informed consent? {26a}

Trial investigators (endoscopists registered under the approved study protocol at each center) will provide participants with a detailed explanation of the study. Participants will be informed of study details in person by the investigator before enrollment, using both verbal and written explanations, and will have the opportunity to ask questions before signing consent. Written informed consent will then be obtained from each participant based on their voluntary decision, using the latest approved version of the consent form. This protocol has received IRB approval from all participating institutions.

### Additional consent provisions for collection and use of participant data and biological specimens {26b}

If the study data are to be used for secondary analyses in future research, additional approval will be obtained from the respective institutional review boards. Participants will be appropriately informed and given the opportunity to provide informed consent or to opt out. The MARBLE trial protocol does not involve the collection or use of biospecimens for research purposes.

## Interventions

### Explanation for the choice of comparators {6b}

EST is currently the standard first-line approach for papillary treatment during the management of small CBDS. As this study aims to evaluate the efficacy of ESBD as a novel alternative, EST is designated as the control intervention, and outcomes will be compared between patients undergoing ESBD and those receiving conventional EST.

### Intervention description {11a}

The procedure will be performed within 14 days of patient enrollment. A standard duodenoscope will be advanced into the duodenum, followed by biliary cannulation using a standard catheter and a 0.025–0.035-inch guidewire. A standard wire-guided sphincterotome will be used to perform EST. In the ESBD group, a minimal incision is made in the 11 to 12 o’clock direction without reaching the hooding fold. In the EST group, a medium incision extending beyond the hooding fold is performed. This standard will be reinforced through pre-study training and photographic documentation to ensure consistency among all operators. In the ESBD group, EPBD is subsequently performed with a diameter matching the lower bile duct (typically 6–10 mm). After completion of these procedures, stone removal will be performed using a retrieval basket and balloon catheter. Compared with conventional EST, ESBD is expected to reduce adverse event risk including bleeding.

### Criteria for discontinuing or modifying allocated interventions {11b}

Allocated interventions may be discontinued or modified after randomization under the following circumstances: if a patient voluntarily requests to withdraw from the study, if findings emerge after enrollment (including during the intervention) indicating that a patient should be excluded, if the study is terminated, or if the investigator determines that discontinuation is appropriate for any other reason. Patients who deviate from the protocol will remain in the intention-to-treat analysis; a per-protocol analysis will also be performed as sensitivity analysis.

### Strategies to improve adherence to interventions {11c}

To ensure adherence to the intervention protocol, all participating endoscopists will be provided with a standardized explanation of the procedural steps during a pre-study meeting. The meeting will serve as an opportunity to align procedural details such as the extent of sphincterotomy and balloon dilation techniques. In addition, study investigators at each site will ensure protocol compliance. Central monitoring and regular communication among centers will help identify and resolve any protocol deviations during the study period.

### Relevant concomitant care permitted or prohibited during the trial {11d}

Concomitant care and interventions are generally permitted at the discretion of the treating physician, provided they do not interfere with the study protocol. The use of antithrombotic agents will be managed according to the Guidelines [5, 6]. Antithrombotic agents may be withheld or adjusted as appropriate based on individual patient risk and in consultation with the prescribing physician.

### Provisions for post-trial care {30}

Participants who experience study-related adverse events will receive appropriate medical care in accordance with the study site’s standard medical practice. No specific ancillary or post-trial care is planned beyond the standard of care routinely provided at each participating institution. Although no specific post-trial care is planned, participants will be informed of the study results upon request, and late adverse events will be monitored during follow-up.

### Outcomes {12}

The primary outcome of this study is the incidence of procedure-related adverse events following endoscopic treatment. Secondary outcomes include the type and severity of adverse events, technical and clinical success rates, total procedure time, time required for stone removal, the need for lithotripsy, and long-term outcomes such as stone recurrence and biliary events during the 5-year follow-up period. The definitions of each outcome, along with their measurement variables, analysis metrics, and time points, are summarized in Table 1.

The definitions and severity grading of procedure-related adverse events will be defined according to the American Society for Gastrointestinal Endoscopy lexicon [7]. Among these, bleeding and pancreatitis are the most anticipated adverse events in this study and represent key safety outcomes; therefore, more detailed criteria are specifically defined for these two events. Intra-procedural bleeding is defined as bleeding that requires hemostatic intervention (excluding preventive measures), while post-procedural bleeding is defined as bleeding confirmed by endoscopy or imaging study such as computed tomography, or inferred from clinical findings such as hematemesis, melena, or a drop in hemoglobin level that cannot be explained by other causes. The diagnosis of pancreatitis is established when at least two of the following three criteria are met: (1) acute abdominal pain and tenderness in the upper abdomen, (2) elevated levels of serum or urinary pancreatic enzymes above the upper limit of normal, and (3) imaging findings indicative of acute pancreatitis [8]. Long-term outcomes, including stone recurrence over 5 years, will be monitored via periodic chart reviews and patient contact; imaging will be performed if clinically indicated.

### Participant timeline {13}

**Fig. 1:**
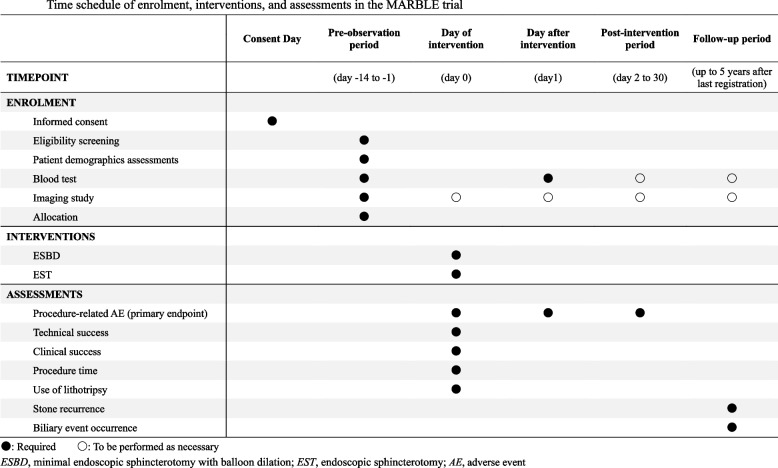
Time schedule of enrolment, interventions, and assessments in the MARBLE trial. ●: Required 〇: To be performed as necessary. *ESBD*, minimal endoscopic sphincterotomy with balloon dilation; *EST*, endoscopic sphincterotomy; *AE*, adverse event

A schematic diagram of the time course and schedule for participants of the MARBLE trial is illustrated in Fig. 1.

### Sample size {14}

The sample size estimation was based on a two-sided chi-square test. A previous retrospective study comparing ESBD and EST reported that the incidence of procedure-related adverse events was 15.8% in the EST group and 4.4% in the ESBD group [3]. Based on these findings, a sample size calculation was performed using a two-sided test with a significance level (*α*) of 0.05 and a power of 80%, assuming a 1:1 allocation ratio. Accounting for an estimated 10% of ineligible or dropout cases, the final target sample size is set at 280 patients (140 in each group). In addition to the primary sample size estimation based on retrospective data, a sensitivity analysis will be performed using alternative event rates reported in other studies to ensure the robustness of the calculation.

### Recruitment {15}

To ensure adequate participant enrollment and achieve the target sample size, this trial will be conducted as a multicenter study involving five high-volume tertiary care hospitals in the Tokai region of Japan. Each participating center has extensive experience in endoscopic management of CBDS and a sufficient case volume to support enrollment. Regular investigator meetings will be held to monitor recruitment progress, identify enrollment barriers, and implement corrective measures as needed. Eligibility screening logs will document the number of patients screened, reasons for non-participation, and reasons for exclusion to improve transparency.

## Assignment of interventions: allocation

### Sequence generation {16a}

After patient enrollment, randomization will be performed using the UMIN INDICE cloud system. A computer-generated allocation sequence will assign participants in a 1:1 ratio to either the ESBD or EST groups. Stratified randomization will be applied based on two allocation factors: (1) presence or absence of pre-procedural biliary drainage, and (2) use or non-use of antithrombotic agents. Within each stratum, variable block sizes will be used to maintain allocation concealment. To ensure allocation concealment and reduce the predictability of the random sequence, details of any randomization restrictions will be maintained in a separate document inaccessible to investigators involved in participant enrollment or group assignment.

### Concealment mechanism {16b}

The allocation sequence will be implemented using the secure, centralized UMIN INDICE cloud-based randomization system. This system ensures allocation concealment by preventing investigators and study personnel from accessing the randomization sequence in advance. Participants will be randomized only after completing the eligibility confirmation and registration procedures, thereby maintaining strict concealment until assignment.

### Implementation {16c}

The computer-generated allocation sequence will be developed and managed by the UMIN INDICE system. Investigators at each participating center will be responsible for enrolling eligible participants. Once enrollment is completed, the system will automatically assign participants to either the ESBD or EST group according to the pre-programmed randomization algorithm.

## Assignment of interventions: blinding

### Who will be blinded {17a}

This is an open-label trial; therefore, neither participants nor treating endoscopists will be blinded to group allocation. Outcome assessors and data analysts will also not be blinded due to the nature of the intervention. However, to minimize bias in outcome evaluation, procedure-related adverse events will be classified and graded according to predefined criteria based on the American Society for Gastrointestinal Endoscopy lexicon, and all relevant outcomes will be objectively assessed using standardized metrics.

### Procedure for unblinding if needed {17b}

As this is an open-label trial, unblinding procedures are not applicable. All participants and treating physicians will be aware of the assigned interventions at the time of treatment.

## Data collection and management

### Plans for assessment and collection of outcomes {18a}

At each participating site, the principal investigator or co-investigators will collect baseline characteristics, procedural data, and outcomes from medical records and procedural findings. These data will be entered into the UMIN INDICE cloud system and stored as electronic case report forms. Each institution will prepare and maintain a separate linkage file that connects the study identification code with the individual participant, ensuring that personal identifiers are not used during data entry. All data queries or communications between the data management team or study office and the participating centers will be handled using only the study ID codes, in order to maintain confidentiality.

To ensure data quality, all investigators will receive instructions in advance regarding standardized definitions of each outcome and data entry procedures. Investigators will undergo training sessions and certification in data entry and outcome assessment procedures before trial initiation. Outcome assessment, including adverse event classification and severity grading, will follow predefined criteria based on the American Society for Gastrointestinal Endoscopy lexicon. The study does not involve patient-reported outcomes or questionnaire-based assessments.

### Plans to promote participant retention and complete follow-up {18b}

To ensure comprehensive follow-up and maximize participant retention, all patients will undergo clinical and laboratory evaluations on the day following the endoscopic procedure. Imaging studies such as computed tomography will be performed as clinically indicated.

Although no specific follow-up protocol is mandated during the post-treatment observation period, patients presenting with symptoms suggestive of adverse events will promptly undergo appropriate blood and imaging tests, following the same assessment principles used on the day after the procedure.

Long-term follow-up will continue until either the participant’s death or the end of the study’s follow-up period (i.e., 5 years after the final participant’s enrollment), whichever occurs first. Data collection during follow-up may include chart reviews, records from other institutions, or telephone interviews, as appropriate. Every effort will be made to obtain outcome data at the end of the study period whenever possible. A loss-to-follow-up rate of less than 10% will be targeted. To achieve this, we will maintain regular contact with participants through scheduled telephone calls and coordinate with referring hospitals for updated information. If the loss-to-follow-up rate approaches 10%, additional measures such as contacting family members or caregivers will be implemented.

### Data management {19}

All study data will be entered into the UMIN INDICE cloud-based data management system by investigators at each participating site. Data will be entered using anonymized study identification codes, and personal identifiers will not be included in the system to ensure confidentiality. Each site will securely maintain a linkage file to connect study IDs with individual participants. The data manager will also screen for missing or unplausible data and ask the corresponding investigator at each center for a data check using study ID codes only. Audit trails will be maintained to track all data modifications. Access to the data system will be restricted to authorized personnel with individual login credentials.

### Confidentiality {27}

All personal information will be handled in accordance with relevant privacy laws and institutional guidelines. Data handling will comply with the General Data Protection Regulation where applicable, in addition to local privacy laws. Participants will be assigned anonymized identification codes at the time of enrollment, and all study data will be managed using these codes. Personally identifiable information will not be included in study databases and will be securely stored at each site. Data sharing between institutions will be conducted using only anonymized codes to ensure confidentiality before, during, and after the trial.

### Plans for collection, laboratory evaluation, and storage of biological specimens for genetic or molecular analysis in this trial/future use {33}

This study does not involve the collection, storage, or laboratory evaluation of biological specimens for genetic or molecular analysis. No biospecimens will be collected for current or future research purposes.

## Statistical methods

### Statistical methods for primary and secondary outcomes {20a}

For the primary outcome, group comparisons of categorical variables will be performed using Pearson’s chi-square test or Fisher’s exact test, as appropriate. A two-sided *p*-value of less than 0.05 will be considered statistically significant.

For secondary outcomes, categorical variables will also be analyzed using Pearson’s chi-square test or Fisher’s exact test. Continuous variables will be compared using the *t*-test or the Mann–Whitney *U* test, depending on the distribution. Normality of continuous variables will be assessed using the Shapiro–Wilk test before applying the *t*-test. Time-to-event variables will be analyzed using the log-rank test, and survival curves will be constructed using Kaplan–Meier estimates. To examine factors associated with each event, multivariable analyses such as linear regression, logistic regression, and Cox proportional hazards models will be applied with covariate adjustment. For multiple subgroup analyses, Bonferroni correction will be applied to adjust the p-value threshold to control the family-wise error rate. If the proportion of missing data exceeds 10%, multiple imputation will be conducted as a sensitivity analysis in addition to the available-case analysis. A two-sided *p*-value < 0.05 will be considered statistically significant for all analyses. All analyses will be performed using R.

### Interim analyses {21b}

No interim analysis is planned for this study. Analyses of short-term and long-term outcomes will be conducted separately in accordance with the predefined statistical analysis plan.

### Methods for additional analyses (e.g., subgroup analyses) {20b}

Subgroup analyses will be conducted based on the following variables: sex, age, body mass index, performance status, comorbidities, presence of liver cirrhosis or renal failure, history of acute pancreatitis, use of antithrombotic agents (none, antiplatelet agents, anticoagulants), platelet count, PT-INR, presence of cholangitis, gallbladder, or gallstones, common bile duct diameter, size and number of bile duct stones, endoscopist’s ERCP experience (< 200 or  ≥ 200 procedures), presence of periampullary diverticulum, use of rectal NSAIDs, pancreatic duct guidewire placement, and placement of a pancreatic stent.

### Methods in analysis to handle protocol non-adherence and any statistical methods to handle missing data {20c}

The primary analysis will follow the intention-to-treat principle, including all randomized participants in the groups to which they were assigned, regardless of protocol adherence. A per-protocol analysis may be performed as a sensitivity analysis to assess the robustness of the findings.

For missing data, an available-case analysis will be used as the primary approach. If the extent or pattern of missing data is substantial, appropriate methods such as multiple imputation may be considered in sensitivity analyses.

### Plans to give access to the full protocol, participant level-data and statistical code {31c}

The full study protocol and statistical code will be made available upon reasonable request to the corresponding author. Access to de-identified participant-level data may also be considered for academic purposes, subject to approval by the study steering committee and in compliance with applicable data protection regulations.

## Oversight and monitoring

### Composition of the coordinating centre and trial steering committee {5d}

The coordinating center for this trial is based at Aichi Medical University, which will oversee the day-to-day management of the study. Responsibilities include organizing investigator meetings, managing case report forms and queries, ensuring data quality and protocol adherence across participating sites, and serving as the main point of contact for regulatory and ethical matters.

A trial steering committee composed of senior investigators from participating institutions will provide overall oversight of the study. The committee is responsible for reviewing study progress, addressing protocol deviations, ensuring scientific integrity, and resolving operational issues. The committee will meet online or on site approximately every 6 months or more frequently as needed.

### Composition of the data monitoring committee, its role, and reporting structure {21a}

A data monitoring committee will not be established for this study. No independent data monitoring committee is planned due to the nature of the intervention, which involves established procedures with low anticipated risk. This decision is based on the nature of the trial, which involves well-established endoscopic procedures with a relatively low risk profile and a short treatment duration. All interventions are delivered during a single procedure, and participants will be closely monitored during the immediate post-treatment period.

Instead, safety and data quality will be overseen by the trial steering committee and the coordinating center. These bodies will regularly review adverse events and the study progress. Any serious adverse events or protocol deviations will be promptly reported and discussed among the investigators.

### Adverse event reporting and harms {22}

All adverse events occurring after the initiation of the protocol-defined intervention will be collected and assessed by the site investigators. Procedure-related adverse events will be defined and graded according to the American Society for Gastrointestinal Endoscopy lexicon, which provides standardized criteria for type and severity.

Adverse events will be documented based on clinical findings, laboratory test results, and imaging as appropriate. Serious adverse events (SAEs) include events that result in death, are life-threatening, require hospitalization or its prolongation, or cause persistent or significant disability, and will be reported promptly to the coordinating center within 24 h of site awareness. SAEs must be reported by the site to the principal investigator, who will notify the ethics committee via the designated reporting system. Shared information on SAEs will be communicated to all participating sites, and appropriate actions will be taken according to each institution’s procedures.

### Frequency and plans for auditing trial conduct {23}

No formal independent audit is planned for this study. However, the coordinating center will regularly monitor trial conduct through central review of data entries, protocol adherence, and adverse event reporting. Any protocol deviations or data discrepancies will be addressed collaboratively with the site investigators.

### Plans for communicating important protocol amendments to relevant parties (e.g. trial participants, ethical committees) {25}

All significant protocol amendments, including changes to eligibility criteria, outcomes, or analysis methods, will be promptly communicated to all relevant parties. These include the ethics committees of participating institutions, trial investigators, the registry, and regulatory authorities as required. If amendments affect participant rights or safety, trial participants will be informed and re-consented as appropriate. All changes will be documented with version control and the date of approval.

### Dissemination plans {31a}

The findings of this trial will be disseminated through presentations at national and international scientific conferences and published in peer-reviewed medical journals to maximize the chances of dissemination of the results to healthcare professionals and the public, and to contribute to improvements in public health. Summary results will also be reported in the jRCT registry in accordance with applicable regulations. Upon request, participants may receive a summary of the study findings in lay-language after study completion.

## Discussion

This multicenter, randomized controlled trial (the MARBLE trial) addresses an important clinical question regarding the optimal papillary treatment for small CBDS. While EST remains the standard first-line technique, ESBD has emerged as a potentially safer and more effective alternative. Retrospective studies have suggested that ESBD may reduce adverse events and procedural burden; however, high-quality prospective data is currently lacking.

The trial presents several practical and operational challenges. Given that all participating centers are tertiary-care hospitals with experienced endoscopists, standardization of technique is expected to be feasible. However, variations in operator experience, especially involving trainees, may influence outcomes, which will be evaluated through subgroup analyses. Furthermore, long-term follow-up extending up to five years poses challenges in maintaining patient retention, especially in the absence of mandated in-person visits; thus, telephone follow-up and remote data collection will be utilized when appropriate. Moreover, the findings of this trial will primarily be applicable to patients with small CBDS (< 12 mm) and no history of prior papillary intervention. While this trial is conducted in high-volume tertiary-care hospitals in Japan, the generalizability of findings to community or lower-resource settings remains uncertain. Further studies are warranted to assess the safety and efficacy of ESBD in diverse practice environments and in patients with larger stones or in those with previous papillary treatments.

Despite these anticipated challenges, this trial is expected to contribute robust evidence to inform clinical decision-making. If ESBD is shown to be superior to EST in reducing procedure-related adverse events, it may become a new standard technique for managing small CBDS. The study will also generate valuable insights into long-term outcomes such as stone recurrence and biliary events, helping shape future practice guidelines.

### Trial status

The current version of the protocol is 1.1, which was updated on 21 February 2025. The recruitment started on 21 April 2025 and is scheduled to be completed on 31 March 2027.

## Data Availability

Access to the final trial dataset will be restricted to the principal investigator and designated members of the study team. There are no contractual agreements that limit investigators’ access to the data. De-identified datasets may be shared with external researchers upon reasonable request and with appropriate ethical approval, in accordance with applicable data protection regulations.

## Abbreviations

CBDS: Common bile duct stone
EST: Endoscopic sphincterotomy
EPBD: Endoscopic papillary balloon dilation
ESBD: Minimal endoscopic sphincterotomy with balloon dilation
SAE: Serious adverse event
